# Regional, sex, and age inequities in asthma hospital admissions in Spain and Portugal

**DOI:** 10.1002/clt2.12349

**Published:** 2024-03-30

**Authors:** Rafael José Vieira, Ana Margarida Pereira, Luís Taborda‐Barata, Frederico S. Regateiro, Manuel Marques‐Cruz, Carlos Robalo Cordeiro, Cláudia Chaves Loureiro, Ignacio J. Dávila, Jean Bousquet, João A. Fonseca, Bernardo Sousa‐Pinto

**Affiliations:** ^1^ Department of Community Medicine, Information and Health Decision Sciences (MEDCIDS) Faculty of Medicine University of Porto Porto Portugal; ^2^ Centre for Health Technology and Services Research Health Research Network (CINTESIS@RISE) Faculty of Medicine of the University of Porto Porto Portugal; ^3^ Allergy Unit CUF Porto Hospital & Institute Porto Portugal; ^4^ PaCeIT – Patient Centered Innovation and Technologies Center for Health Technology and Services Research (CINTESIS) Faculty of Medicine University of Porto Porto Portugal; ^5^ UBIAir ‐ Clinical & Experimental Lung Centre and CICS‐UBI Health Sciences Research Centre University of Beira Interior Covilhã Portugal; ^6^ CICS – Health Sciences Research Centre University of Beira Interior Covilhã Portugal; ^7^ Department of Immunoallergology Cova da Beira University Hospital Centre Covilhã Portugal; ^8^ Allergy and Clinical Immunology Unit Centro Hospitalar Universitário de Coimbra Coimbra Portugal; ^9^ Institute of Immunology Faculty of Medicine University of Coimbra Coimbra Portugal; ^10^ Center for Innovative Biomedicine and Biotechnology (CIBB) Faculty of Medicine University of Coimbra Coimbra Portugal; ^11^ Department of Pulmonology University Hospital of Coimbra Coimbra Portugal; ^12^ Faculty of Medicine University of Coimbra Coimbra Portugal; ^13^ Allergy Service University Hospital Salamanca Spain; ^14^ School of Medicine University of Salamanca Salamanca Spain; ^15^ Spanish Society of Allergology and Clinical Immunology Salamanca Spain; ^16^ Institute for Allergology Charité ‐ Universitätsmedizin Berlin Corporate, Member of Freie Universität Berlin and Humboldt‐Universität zu Berlin Berlin Germany; ^17^ Allergology and Immunology Fraunhofer Institute for Translational Medicine and Pharmacology ITMP Berlin Germany; ^18^ ARIA Montpellier France

## Abstract

**Background:**

Asthma presents a significant health challenge, imposing a considerable burden on healthcare services. Discrepancies in asthma‐related hospitalisations may reflect underlying health disparities. We aimed to analyse inequities in asthma hospital admissions in mainland Portugal and Spain, from a regional perspective and considering sex and age.

**Methods:**

We conducted a retrospective study using data from the Spanish and Portuguese national hospitalisations databases. We calculated crude national and regional yearly hospitalisation rates according per Nomenclature of Territorial Units for Statistics region. Additionally, we calculated hospitalisation rates adjusted for asthma prevalence and the female‐to‐male ratio in asthma hospital admissions per age group, considering the female‐to‐male ratio in the overall population.

**Results:**

Between 2012 and 2016, there were 92,084 asthma hospital admissions in mainland Spain and 7717 in mainland Portugal. There was a trend for a higher‐than‐average rate of asthma‐related hospitalisations in the Northern regions of both countries. Women had a hospitalisation rate that was 3.2 times higher than men. Age was associated with higher risk for asthma hospitalisation, with individuals aged 65 and older displaying a hospitalisation rate 4.5 times higher than those under 65. Additionally, while hospitalisations in women aged <65 years were 2.3 times more likely than in men of the same age, hospitalisations in women aged ≥65 years were 3.5 times higher than in men aged ≥65 years.

**Conclusion:**

This study suggests that marked regional inequities in asthma hospital admissions exist in Spain and Portugal. Additionally, women are particularly at risk of hospitalisation due to asthma, and such risk increases with age.

To the editor,

Asthma is a highly prevalent disease with a high burden for healthcare services. In the Iberian Peninsula, average asthma costs for an adult patient are estimated to be €708 per year in Portugal and €1726 in Spain.^1^, ^2^ Asthma is an ambulatory care‐sensitive condition, as proper management has been shown to avoid exacerbations and decrease hospitalisations.^3^ Therefore, disparities in asthma hospitalisations may reflect underlying health inequities. A previous study has shown regional health inequities in asthma hospital admissions in Portugal,^4^ but regional inequities in Spain have not been reported (although an analysis is available for the Asturias region^5^). Additionally, possible inequities related to sex and age were not assessed. This study aimed to analyse inequities in asthma hospital admissions in mainland Portugal and Spain, from a regional perspective and considering sex and age‐related factors.

We conducted a retrospective observational study using data from the Spanish and Portuguese national hospitalisations databases. The Spanish database contains data from all private and public hospitals, while the Portuguese database contains data from all public hospitals (accounting for 85% of Portuguese hospitalisations).^6^, ^7^ We included all inpatient episodes of patients aged ≥20 years with a main diagnosis of asthma (ICD‐9‐CM code 493.x or ICD‐10‐CM code J45.x) from 2012 to 2016. For each episode, we had data on patients' age, sex and third level of Nomenclature of Territorial Units for Statistics (NUTS3) of residence. We calculated crude national and regional yearly hospitalisation rates according per NUTS3 region. In addition, we computed hospitalisation rates adjusted for asthma prevalence. For Spain, we used prevalence data from the European Health Interview Survey, which reports data at the NUTS2 level (to calculate prevalence‐adjusted hospitalisation rates per Spanish NUTS3 region, we assumed the prevalence to be homogeneous in all NUTS3 within each NUTS2 region) (https://www.ine.es/jaxiT3/Tabla.htm?tpx=47663). For Portugal, we used unpublished data on asthma prevalence per NUTS3 region from the Portuguese National Asthma Survey.^8^ Since different coding practices may lead to systematic differences in asthma hospitalisation rates in Spain and Portugal, we calculated the crude and prevalence‐adjusted national average, and, for each NUTS3, we calculated the ratio between the regional hospitalisation rate and the national average. Additionally, we computed the female‐to‐male ratio in asthma hospital admissions per year of age, adjusted for the female‐to‐male ratio in the overall population. Demographic data (resident population per region overall and by age and sex) were obtained from the Portuguese and Spanish National Statistics Institutes.

From 2012 to 2016, there were 92,084 asthma hospital admissions in mainland Spain and 7717 in mainland Portugal. Women accounted for 78% and 73% of the hospital admissions in Spain and Portugal, respectively. The median age at hospital admission was 70 (interquartile range [IQR] = 29) years in Spain and 61 (IQR = 29) years in Portugal. On average, there were 50.6 hospitalisations/100,000 inhabitants in Spain and 19.4/100,000 in Portugal. In Portugal, marked national disparities in the rate of asthma hospitalisations were observed, with some NUTS3 regions in the Centre displaying hospitalisation rates at least twice that of the national average. In Spain, the regions with the highest hospitalisation rates were those of Asturias (137.8/100,000) and Alava (112.1/100,000). A trend for a higher‐than‐average rate of hospitalisations due to asthma was noticeable in the Northern regions of both Spain and Portugal, with six Northern regions (five in Spain and one in Portugal) displaying a rate of hospitalisations that was at least 1.5 times the national average (Figure 1A). When accounting for the prevalence of asthma in NUTS3 regions, regional disparities are accentuated (Figure 1B).

**FIGURE 1:**
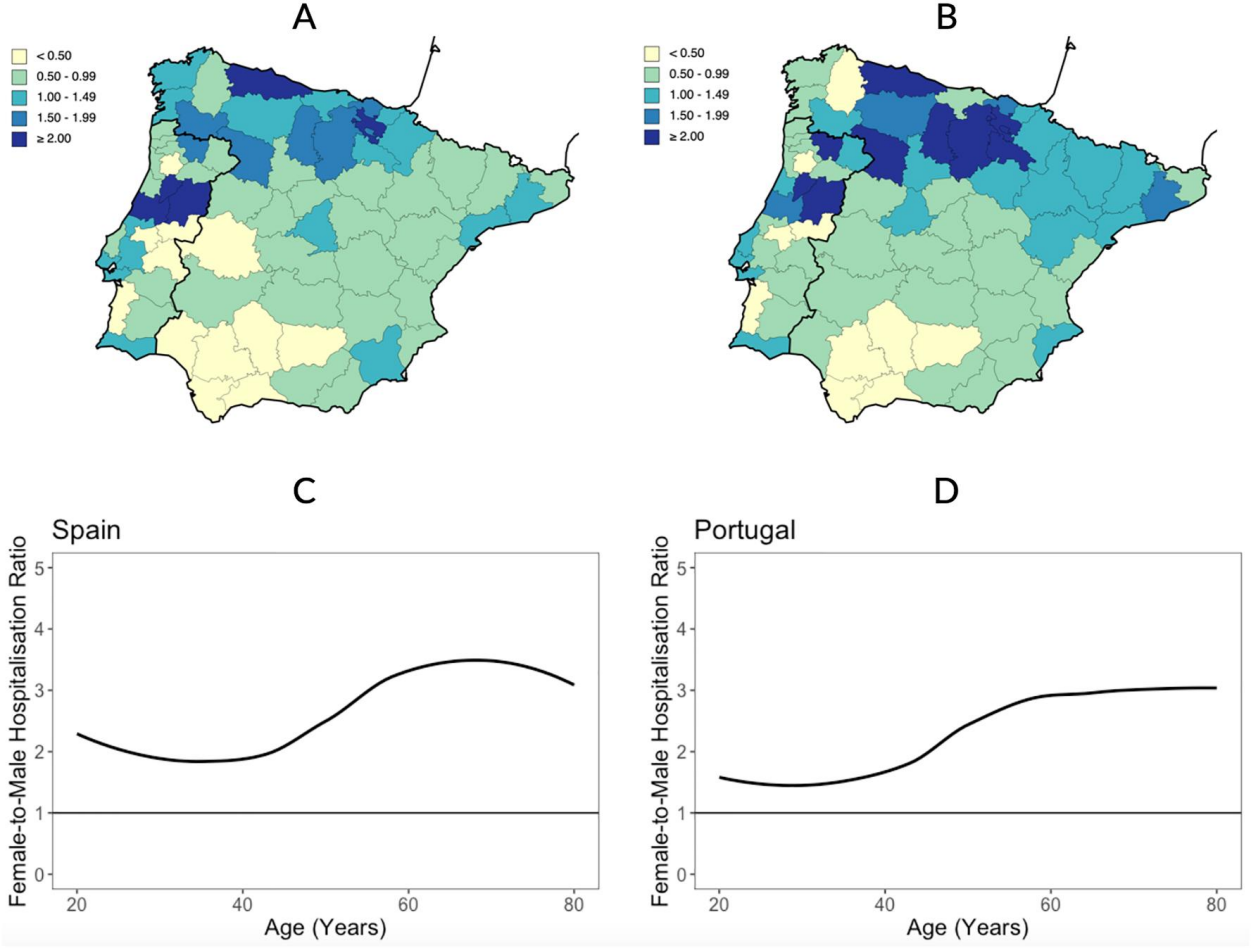
Panels (A and B) show crude (Panel A) and prevalence‐adjusted (Panel B) hospital admissions per NUTS 3 in mainland Spain and Portugal, as ratios compared to the national annual average. Panels (C and D) show the female‐to‐male ratio of hospital admissions in Spain (Panel C) and Portugal (Panel D), adjusted for the female‐to‐male population ratio.

Considering Spain and Portugal, women had a hospitalisation rate that was 3.2 times higher than men. Age was also found to associate with higher risk for asthma hospitalisation, as people aged 65 years and older displayed a hospitalisation rate that was 4.5 times higher than people aged <65. Additionally, while hospitalisations in women aged <65 years were 2.3 times more likely than in men of the same age, hospitalisations in women aged ≥65 years were 3.5 times higher than in men aged ≥65 years. This age‐increasing female‐to‐male hospital ratio admissions in Spain and Portugal (Figure 1C,D) shows that not only were hospitalisations due to asthma more frequent in older patients, but age was found to accentuate sex‐inequities in asthma hospital admissions (even after adjusting for the number of females and males at risk of hospitalisation).

There are some limitations to this study. Firstly, we relied on the quality of diagnoses and clinical coding. Some of the differences observed in hospitalisation rates in Spain and Portugal may be related to different coding practices, and there may be information biases related to miscoding. To partially solve this issue, we calculated the ratio of regional hospitalisation rates to the national average. Additionally, prevalence data was not available at NUTS3 level for Spain. However, it is unlikely that differences in prevalence between NUTS3 regions within each NUTS2 region considerably affects our results, as NUTS3 in Spain correspond to provinces, which—within each autonomous community (NUTS2)—are not expected to differ widely from each other on asthma prevalence. Finally, since this study is based on a secondary analysis of administrative nationwide data, we were unable to account for or study potential confounders.

In conclusion, our study shows marked regional inequities in asthma hospital admissions, with Northern regions in the Iberian Peninsula displaying a higher rate of asthma hospital admissions compared with the national averages. Additionally, we found women to be particularly at risk of hospitalisation due to asthma, and that such risk increases with age, as had previously been reported in other epidemiological studies.^9^ Actions should be taken to improve care to decrease regional, sex, and age disparities in asthma care in the Iberian Peninsula, possibly involving an identification and closer monitoring of patients at increased risk of hospitalisation. For those purposes, local care structures (e.g., primary care and social assistance), comprehensive electronic registries and digital patient‐centred tools may be particularly useful.

## AUTHOR CONTRIBUTIONS

Rafael José Vieira participated in data analysis and manuscript writing. Bernardo Sousa‐Pinto participated in study design and manuscript writing. Ana Margarida Pereira, João A. Fonseca and Jean Bousquet participated in study design and critical revision of the manuscript. All remaining authors participated in manuscript writing and critical revision of the manuscript.

## CONFLICT OF INTEREST STATEMENT

The authors declare no conflicts of interest.

## FUNDING INFORMATION

Portuguese National Funds and Community Funds from the European Social Fund and Programa Por_Norte through Fundação para a Ciência e a Tecnologia, 2022.12787.BD

## Data Availability

The data that support the findings of this study are available from the corresponding author upon reasonable request.
